# The Dental Rubber Cases—A Novel Law Suit

**Published:** 1874-01

**Authors:** 


*The Dental Rubber Cases—A Novel Law Suit.*—From the *N. Y. Herald* we take the following, to which reference is made in the selected article in the present No. of the *Journal:*

The Goodyear Dental Vulcanite Company, owners of the patent for hard rubber plates for artificial teeth, have just instituted a suit in the United States Circuit Court, in this city, against Samuel S. White, of Philadelphia, laying their damages at $100,000, the alleged offence complained of being what is known in law as "maintenance," which is defined in the books as "intermeddling in a suit that no way belongs to one by maintaining or assisting either party with money or otherwise to prosecute or defend it."

Mr. White is an extensive manufacturer of materials, which are purchased by dentists for their practice. The dentists are opposed to the payment of royalties to the company, which owns the patent, and it is alleged that Mr. White prepared defences which, it is claimed, he supposes the dentists might make against the Company when called on to pay for their license; that he voluntarily furnished at his own expense such defences, in a printed form, to the licensees of the Company and to others, and that he payed the defendants' expenses of law suits instituted by the Company against those who have followed his advice. The Company claim, that their losses by the alleged interference of Mr. White are not less than $100,000.

The other side remains to be heard, and when Mr. White's answer has been put in it will be duly noted.

